# Race, ethnicity, ancestry, and aspects that impact HLA data and matching for transplant

**DOI:** 10.3389/fgene.2024.1375352

**Published:** 2024-03-15

**Authors:** Abeer Madbouly, Yung-Tsi Bolon

**Affiliations:** Center for International Blood and Marrow Transplant Research (CIBMTR), Minneapolis, MN, United States

**Keywords:** race, ethnicity, ancestry, HLA, HCT, solid organ transplant, donor registry

## Abstract

Race, ethnicity, and ancestry are terms that are often misinterpreted and/or used interchangeably. There is lack of consensus in the scientific literature on the definition of these terms and insufficient guidelines on the proper classification, collection, and application of this data in the scientific community. However, defining groups for human populations is crucial for multiple healthcare applications and clinical research. Some examples impacted by population classification include HLA matching for stem-cell or solid organ transplant, identifying disease associations and/or adverse drug reactions, defining social determinants of health, understanding diverse representation in research studies, and identifying potential biases. This article describes aspects of race, ethnicity and ancestry information that impact the stem-cell or solid organ transplantation field with particular focus on HLA data collected from donors and recipients by donor registries or transplant centers.

## Introduction

Race, ethnicity, and ancestry are terms often confused and used by many interchangeably. However, each of these terms has a distinct meaning. **Race** is a dynamic, and complex social construct, generally applied to a group of individuals based on **observed biological or phenotypic traits,** where these characteristics have acquired socially significant meaning (such as Black or White). **Ethnicity** is a socially constructed concept employed to refer to groups of individuals who share a similar **cultural heritage or identity** (history, language, and/or religion), where these characteristics have social meaning (such as Hispanic). In contrast, **Ancestry** refers to ancestral origin, an individual’s lineage of descent, or the **geographic history of an individual’s ancestors according to signatures in their DNA** (Borrell et al., 2021; Lu et al., 2022).

Ancestry can be defined geographically, genealogically, or genetically, and can suffer limitations like race or ethnicity. Geographic ancestry refers to ancestors originating from similar geographic regions (such as South Asian). Genealogical ancestry refers to one’s ancestral pedigree (family tree), and genetic ancestry refers to ancestors from whom one is biologically descended (for example, if segments from an individual’s genome are found similar to individuals from particular continental groups like European or African). While the construct of race relies on perceived physical characteristics like skin color, ethnicity also captures elements of an individual’s identity beyond the physical. As a result, the use of each term is subject to various interpretations by researchers, study participants, and readers. For example, the terms “Asian” and “Hispanic” are interpreted differently in different regions of the world. Additionally, several race groups can themselves include multiple ethnic groups: for example, Black, White, and Asian races can all include individuals belonging to Hispanic and/or Jewish ethnicities (Lu et al., 2022).

These terms are important not just for social purposes, but for numerous medical applications, including matching for solid organ and stem-cell transplantation. The term “matching” refers to identifying the similarities of the patient and stem-cell or solid-organ donor of a gene system called Human Leukocyte Antigen (HLA). These are genes in the major histocompatibility complex (MHC) region on chromosome 6 that help code for proteins differentiating between self and non-self and play a significant role in the body’s immune defense. Because of the nature of HLA function, these genes are extremely polymorphic with thousands of alleles already identified (Robinson et al., 2016; Robinson et al., 2020; Barker et al., 2023) and have been under immune selective pressure in humans for thousands of years (Prugnolle et al., 2005; Chang and Ferrone, 2007). This means that the different immune responses that help humans survive pathogens, migration patterns, wars, colonization movements, natural catastrophes, or changes in procreation patterns in different areas of the world have helped shape the HLA alleles and haplotypes (alleles on the same chromosome) in every region, depending on the selective pressures at play. For example, as generations of African humans survived Malaria, some individuals of African ancestry carry HLA alleles that are known to be resistant to Malaria such as HLA-B*53:01 and HLA-C*06:02 (Digitale et al., 2021). There are numerous other examples of population-specific HLA associations with disease or adverse drug reactions such as the association of HLA-DRB1*04:02 and HLA-DQB1*03:02 in Jewish populations with the autoimmune disease Pemphigus vulgaris (Drenovska et al., 2022) and the adverse reaction to the drug Carbamazepine in individuals of East and South-East Asian ancestry carrying HLA-B*15:02 (Chen et al., 2011). Accordingly, *the concept of geographical ancestry (ancestors originating from similar geographic regions) could be a differentiator of HLA genotypes*.

Additionally, some worldwide populations have been exposed to several patterns of admixture resulting in the emergence of new populations. For example, individuals identifying with the African American race have an African and European ancestral admixture while individuals identifying with Hispanic ethnicity have multiple levels of Indigenous-American, European, and African ancestral admixtures (Bryc et al., 2015). These migration-driven admixtures further increase HLA diversity and contribute to shaping HLA alleles and haplotypes in these regions. For example, an HLA genotype (2 haplotypes) can be common in African or European populations, but when a new genotype is formed with one haplotype from each continental group, the new genotype may not be common at all. All these factors contribute to the formation of certain HLA patterns particular to each geographical area in the world. It is for these reasons that we collect self-identified race and ethnicity (SIRE) data from volunteer donors joining stem-cell registries in general.

Collecting SIRE data is a cost-effective way, *in lieu* of the more costly genetic ancestry analysis, to stratify registry members into different populations. Without this population information it is difficult to perform multiple processes on the donor registry, including imputation to fill data gaps and resolve data ambiguities (Madbouly et al., 2014; Maiers et al., 2019; Israeli et al., 2023), finding potentially matched donors to patients (Dehn et al., 2016) or reporting match rates to government-affiliated organizations (Gragert et al., 2014a). Even if funds were available for some registries to perform genetic ancestry analyses (Bryc et al., 2015), the quality of the estimated genetic admixture results are heavily dependent on the sample size and structure of the underlying reference data used to estimate these admixture proportions. Individuals belonging to populations underrepresented in these reference datasets may receive inaccurate admixture calculations due to biases introduced by unbalanced reference datasets. The problem is exacerbated for individuals of mixed ancestry. Collecting SIRE information, even if some inaccuracies are involved with self-identification, remains useful for representing multiple human populations in medical settings, particularly if paired with geographical ancestry information (Hollenbach et al., 2015; Lu et al., 2022).

The remainder of this article describes different applications of SIRE data in solid organ or stem-cell transplantation, with primary focus on different stages of analyzing HLA data as applied by different teams in the NMDP^®^ stem-cell donor registry.

### The importance of donor and patient SIRE information

Donor and patient race and ethnicity data impact multiple aspects of the stem-cell or solid-organ donor registry operations, particularly the HLA matching process for Hematopoietic Cell Transplantation (HCT). To appreciate the impact of SIRE data on stem-cell registry operations, an understanding of HLA haplotype frequencies (Kollman et al., 2007; Gragert et al., 2013; Gragert et al., 2023) and related processes is necessary. For decades, donor/recipient HLA matching has been crucial for the success of solid organ and stem-cell transplantation (van Rood, 2000; Sheldon and Poulton, 2006; Mahdi, 2013; Dehn et al., 2019). However, genotyping methodologies (Erlich, 2012; Edgerly and Weimer, 2018), HLA genes/loci relevant to transplant success (Dehn et al., 2019) and how HLA data are communicated and reported between different transplant hubs have evolved over the decades. To keep up with this changing landscape, donor registries continuously developed algorithms to accommodate changes in genotyping technologies and clinical practice. In the early days of stem-cell transplantation, only *HLA-A* and *HLA-B* were tested for donor/recipient matching. Following clinical research that showed the favorable impact of matching *HLA-DRB1*, then *HLA-C*, then *HLA-DQB1* and most recently *HLA-DPB1* on transplant outcomes (Ruggeri et al., 2023), registries continued to screen registry members for more HLA loci. Most major donor registries currently genotype nine HLA loci at donor recruitment; *HLA-A, -B, -C, -DRB1, -DRB3/4/5, -DQB1, -DQA1, -DPA1 and -DPB1* (*HLA-DRB3, DRB4* and *DRB5* genes behave as alleles of a single locus (Bodmer, 1984; Nomenclature for factors of the HLA system, 1988; Fernández-Viña et al., 2013)). However, much of the NMDP registry HLA data (and potentially other registries) is still missing most of these loci. For registries to be able to provide match predictions for five or more HLA loci, statistical imputation methods (Gourraud et al., 2005; Madbouly et al., 2014) are applied to fill these gaps and resolve any ambiguities reported in the data. On the solid-organ side, some transplant centers use publicly available imputation tools such as Haplostats^©^ (haplostats.org) (Gragert et al., 2013), developed and maintained by the NMDP Bioinformatics team, to resolve HLA data ambiguities or fill gaps in the data.

Statistical imputation is the process of filling data gaps or resolving data ambiguities using a guided statistical approach that utilizes reference data and a maximum likelihood or other statistical approach. Imputation of HLA data (Madbouly et al., 2014) utilizes a maximum likelihood approach and reference data from population haplotype frequencies (Gragert et al., 2013; Gragert et al., 2023) generated via the Expectation Maximization (EM) algorithm (Single et al., 2002; Kollman et al., 2007). Reference population-specific datasets are important for the accuracy of imputation predictions since HLA is closely linked to the evolutionary history of human populations. While an HLA allele may have different frequencies in different populations, it is the haplotypes that are mostly representative of each population (Gragert et al., 2013; Gragert et al., 2023). This is due to the linkage disequilibrium (LD) patterns and the relatively low recombination rate particular to the MHC region (Dawkins and Lloyd, 2019) which leads to the inheritance of HLA alleles in conserved haplotypes (Dawkins and Lloyd, 2019). These “ancestral haplotypes” are more population-specific than alleles and therefore a particular HLA-haplotype could be common in a certain population but not in another. Therefore, knowledge of haplotypes specific to certain populations guides the imputation process to fill the data gaps and resolve data ambiguities by aligning the population of the genotype being imputed with these ancestral haplotypes. This leads to proper alignment of the imputed genotype with the population it is associated with.

A robust and reliable reference dataset must satisfy multiple criteria, such as large samples from each population to represent the diversity in ancestral haplotypes and proper separation of different population sample sets to adequately capture the population genetic characteristics while minimizing admixture with other populations. These conditions are opposing in nature and form a significant populations genetics challenge. A genetically diverse population will have many more haplotypes that occur at smaller frequencies in the population than a relatively homogeneous population with less haplotypes that occur at larger frequencies. Large sample sizes ensure that the majority of relatively common, and some relatively rare haplotypes in the underlying populations are represented in the haplotype frequencies which would improve prediction accuracies for statistical imputation and donor recipient HLA matching.

For example, when structuring reference data to generate haplotype frequencies in a stem cell registry for donors of African ancestry, aggregating HLA genotypes of donors whose ancestors roots come from multiple African regions in a single group can generate a large sample at the expense of combining multiple underlying populations with significant HLA diversity that could have different HLA profiles such as African- American, West African, East African, Nigerian, Somali, Jamaican, etc. This contradicts the conditions required to run the EM algorithm (Single et al., 2002; Kollman et al., 2007) to generate haplotype frequencies and masks the private population details needed to serve specific populations for matching, recruitment, clinical care, and government reporting. In contrast, grouping donor samples by single population can provide valuable information private and specific to a single population and would conform with the Hardy-Weinberg equilibrium conditions mandated by the EM algorithm (Excoffier and Slatkin, 1995), however the sample sizes could be too small to capture the HLA haplotype diversity in the population reference datasets which would lead to generating frequency datasets inadequate for proper imputation and matching. The challenge is to balance the population definition and sample size. At the NMDP donor registry, 21 detailed populations are designed for operational matching (Table 1) (Gragert et al., 2013). These population categories were generated by merging smaller populations that are within close geographic proximity and of genetically similar HLA haplotypes while maximizing sample sizes for these populations to produce reliable haplotype frequency datasets that capture HLA diversity in these populations (Gragert et al., 2013). Reliability of haplotype frequency datasets is determined through an extensive validation process of the frequency data and imputation and matching results to guarantee proper alignment of imputation and match predictions with expectations (Eberhard et al., 2013; Madbouly et al., 2014; Dehn et al., 2016).

**TABLE 1:** Current operational race and ethnic groups at the NMDP registry (Gragert et al., 2013).

Detailed populations	Description	Broad population	Description
AAFA	African American	AFA	African American
AFB	African		
CARB	Black Caribbean		
SCAMB	Black South or Central America		
AINDI	South Asian	API	Asian or Pacific Islander
FILII	Filipino		
HAWI	Hawaiian or other Pacific Islander		
JAPI	Japanese		
KORI	Korean		
NCHI	Chinese		
SCSEAI	Other Southeast Asian		
VIET	Vietnamese		
EURCAU	European Caucasian	CAU	Caucasian
MENAFC	MidEast/No. Coast of Africa		
MSWHIS	Mexican or Chicano	HIS	Hispanic
SCAHIS	South/Cntrl Amer. Hisp.		
CARHIS	Caribbean Hispanic		
CARIBI	Caribbean Indian	NAM	Native American
AMIND	North American Indian		
AISC	American Indian South or Central American		
ALANAM	Alaska Native or Aleut		

Population information used to generate the reference haplotype frequencies are obtained from SIRE data collected during the recruitment process of registry members. The presence of inaccuracies in the collected data may introduce a source of error for frequency estimation. These inaccuracies arise due to different factors such as individuals identifying differently over time, lack of alignment of declared race/ethnicity with genetic ancestry, unknown ancestral roots by adopted individuals, among other reasons. However, the availability of a large sample of individuals in each population can minimize the impact of these inaccuracies (Single et al., 2002).

Match predictions generated by HapLogic^®^ (Dehn et al., 2016), the NMDP matching algorithm, for each patient searching the registry are directly affected by imputation methods. Embedded SIRE–derived population genetics haplotype frequencies are used to enumerate possible patient and unrelated donor or cord blood unit allele-level haplotype pairs and associated likelihoods according to an individual’s HLA typing and SIRE broad and detailed race/ethnic categories (Table 1). We have previously reported matching validation results reporting the performance of the HapLogic matching process for each donor SIRE group. Validation was performed on a large cohort of donor-recipient pairs with varying HLA typing resolutions (Dehn et al., 2016). Overall, HapLogic demonstrated superior performance for predicted HLA matching for all donor SIRE groups. However, some prediction uncertainty was observed for intermediate matching probabilities which could be partially attributed to population substructure, SIRE misreporting, and inability of the algorithm to deal with individuals with recent continental admixture (i.e., parents or grandparents from different ethnic backgrounds) (Dehn et al., 2016). This confirms the importance of accurately collecting and reporting SIRE data due to possible clinical influence.

Other applications that rely on haplotype frequencies include Calculated Panel Reactive Antibody (CPRA) calculations for solid-organ allocation (Kransdorf et al., 2017). Using expanded haplotype frequencies for CPRA calculations, with more expanded SIRE data and larger sample sizes to generate the frequencies, has demonstrated greater accuracies for sensitized candidates compared to a version of the CPRA calculator that used limited SIRE data (Rushakoff et al., 2022).

Like the donor side, the collection of accurate SIRE information from patients is important and can impact multiple aspects of a transplant. One of the most impactful applications of patient SIRE information is diversity in healthcare research in general, and clinical research in particular. There are added challenges in the collection of patient SIRE information such as the significant gaps in Electronic Health records (EHR) (Getzen et al., 2023) and the reporting of SIRE information by third party observer *in lieu* of self-identification (Hasnain-Wynia and Baker, 2006; Luisa et al., 2021; Lu et al., 2022). The validity of race and ethnicity classification may depend on whether it is self-reported by a research participant or patient or assigned by a research assistant or healthcare worker (i.e., observer-classified). An analysis of the US health survey data from the Behavioral Risk Factor Surveillance System found that agreement between self- and observer-identified race varied across racial and ethnic groups. Higher agreement rates existed among self-identified Black (96% agreement) and White (98% agreement) participants, with lower agreement rates among non-Black minority groups (35% agreement among Native Hawaiian and other Pacific Island participants) (Jones et al., 2008). Similar results were obtained from an analysis of the US Veterans Affairs healthcare users (Sohn et al., 2006). Discrepancies between self-reported and observer classification of race and ethnicity can have substantial implications on health research findings. For example, observer-assigned compared to self-identification of race has led to underestimations of infant mortality and cancer incidence of Native Americans (Williams, 1996). For all of the above reasons, it is concluded that self-reported identity is preferred over observer classification (Lu et al., 2022).

An important application of patient and/or donor SIRE is to resolve ambiguities or gaps in HLA genotypes, whether to perform donor/recipient matching for HCT or avoid donor-specific HLA antibodies against donor antigens for mismatched HCTs or solid organ transplants. SIRE information is crucial to the accuracy of imputation results. A detailed imputation example is described in the supplementary document that highlights possible inaccuracies in imputation results that could be introduced by erroneous SIRE input to imputation. In the first scenario, and HLA genotype is input to Haplostats^©^ for imputation without any SIRE input which results in multiple possibilities for the imputed genotype results (Supplementary Figures S1, S2). Because a different reference frequency dataset is used for each population selection, a single haplotype can have a different frequency in each population and therefore the prediction likelihoods may differ in each population as well as the alleles in each imputed genotype.

In the second scenario, SIRE information (AFA) was used in the Haplostats input. The imputation result only shows predictions for the input population (Supplementary Figures S3, S4). The predictions are more concordant with genotypes expected in an AFA population. Importantly, the prediction likelihoods indicate the level of certainty in each of the predicted genotypes. It is crucial not to conflate inaccuracy with uncertainty. Inaccuracies are introduced by ambiguities in the genotyping process. These inaccuracies are reduced and often eliminated by the imputation process that provides a population-specific prediction with a quantified level of prediction certainty.

The imputation example demonstrates the importance of SIRE information for more precise prediction of unambiguous HLA genotypes and other related processes that follow such as virtual crossmatch and molecular mismatch analysis. Importantly, registries do not proceed with donor predictions alone as the true HLA genotypes for potentially matched donors, but rather conduct confirmatory HLA typing prior to transplant. However, imputation can leverage larger potential donor pools and save valuable time and cost in identifying the most compatible donors.

There are numerous other applications for patient SIRE information including informing donor recruitment for patients unable to find HLA-matched stem-cell donors, government reporting, and identifying important social determinants of health to inform effective planning and administration of multiple patient services and grant programs.

Beyond transplant, patient SIRE information within Electronic Medical Records (EMR) is an important resource for numerous clinical research disciplines (Uslu and Stausberg, 2021; Rotenstein et al., 2022). Clinical studies that investigate topics around clinical outcomes (Lee et al., 2007; Madbouly et al., 2017; Sigmund et al., 2022), disease associations (Gragert et al., 2014b; Hachicha et al., 2018; Drenovska et al., 2022; Ma and Kerkar, 2023; Li et al., 2024), adverse drug reactions (Chen et al., 2011; O'Connor and Grissinger, 2014; Stephens et al., 2014), polygenic risk scores (Lewis and Vassos, 2020), genome-wide analyses (Li et al., 2008; Hindorff et al., 2009; Laurie et al., 2010), etc. Require subject cohorts with underlying diversity to reach robust results that can be applied to patients with multiple ancestral roots. However, there are significant gaps and inaccuracies in EMR SIRE information (Getzen et al., 2023; Samalik et al., 2023), particularly for patients of color (Lu et al., 2022) due to numerous reasons including observer-based reporting, mistrust in the healthcare system, and possibly other. The presence of these challenges restricts extending the findings of clinical research to multiple populations which may add more challenges to the healthcare system at large.

### Improving the collection, classification, and quality of SIRE data

#### Evolution of the donor SIRE form at the NMDP registry

At the NMDP donor registry, the collection of SIRE information has evolved over decades of registry operations starting from using just the main broad race groups (prior to 1996) and evolving to more population details for each of the main SIRE groups (1996–2002). In 2002, the NMDP adopted the change mandated by the Office of Management and Budget to consider “Hispanic” an ethnicity rather than a race (Ulmer et al., 2009). This change was applied to all donor and patient registry forms. Since this change was made in effect, continued review of the SIRE categories used to collect this information from members joining the registry identified some areas of improvement, including:

• The need to bring back the Hispanic/Latino detailed categories (e.g., Cuban, Mexican, Puerto Rican, etc.). When these details were removed and replaced by a single category (Hispanic ethnicity), multiple registry members of Hispanic ethnicity could not identify with any of the listed race categories on the form and therefore some of the details on their ancestral background were lost.
• Multiple world regions were not included on the SIRE form adopted in 2002 such as Central Asia, African sub-regions, and some populations in the Oceania region.
• Combining genetically diverse populations under single categories (e.g., African or South Asian) concealed some details that potentially removed information needed to better serve populations of color such as African, Middle Eastern, and South Asian populations.
• The existence of embedded sub-populations within our broad groups masked some HLA details that can help better serve some patients such as members of Jewish ethnicity embedded within the larger European Caucasian group.

Based on this review and extensive prior conducted research, a new, more inclusive donor recruitment form was launched by the NMDP in summer 2020 that addresses the above issues. Table 2 lists the new categories currently used by the NMDP registry to collect SIRE information at donor recruitment.

**TABLE 2:** New race, ethnicity and geographical ancestry categories used for the NMDP donor recruitment form since summer 2020.

Broad category	Detailed category	Broad category	Detailed category
White	Central Asian	Hispanic or Latino	Caribbean Hispanic
	Russian or former Soviet Union		South or Central American Hispanic
	Eastern European		Brazilian
	Southern European		Other Hispanic or Latino
	Northern European	Asian	Chinese
	Western European		Japanese
	White Caribbean		Korean
	White south or central American		Taiwanese
	Other white		Malaysian
Jewish	Ashkenazi		Mongolian
	Sephardi		Filipino
	Mizrahi		Vietnamese
	Other Jewish		Thai
Middle Eastern	North African		Indian
	East Mediterranean		Pakistani
	Arab peninsula		Other Indian subcontinent
	Other Middle Eastern		Other Southeast Asian
Black or African	African American	Native American	Alaskan Native or Aleut
	West African		Indigenous North American Indian
	East African		American Indian/South or Central American
	South African		Caribbean Indian
	Black Caribbean		Other Native American
	Black South or Central American	Pacific Islander	Polynesian
	Other Black or African		Native Hawaiian
Hispanic or Latino	Mexican		Melanesian
	Cuban		Micronesian
	Puerto Rican		Other Pacific Islander

The new categories merge race, ethnicity and geographical ancestry categories based on feedback directly received from registry members, published research, and designed solutions for data issues caused by previous historic forms. Some of the changes included:

- Expanding the broad groups to include Middle Eastern (previously a detailed category) and Jewish (which includes individuals of distinct HLA types) and the terminology was modified to be more inclusive (for example: Hispanic or Latino vs. Hispanic, Black or African vs. Black or African American)
- Adding geographical details under the Hispanic or Latino broad group that were missing in the previous form.
- Dividing the Middle Eastern group into three distinct groups based on published HLA literature (Al-Awwami et al., 2012; Hajeer et al., 2013; Bishara et al., 2019; Alfraih et al., 2021) and internal analysis of registry members.
- Including geographical regions absent in previous forms like Central Asian, East and West African and detailed Pacific Island regions.
- Adding details of Jewish ethnicity to capture the corresponding HLA types.

The new categories captured from the donor recruitment form are internally mapped to the operational detailed and broad groups (Table 1) that are used to generate haplotype frequencies and match donors and patients. Internal validations are underway to investigate expanding current operational SIRE categories using the updated data being collected. Other efforts are underway to expand the patient categories to match the new donor groups in Table 2.

#### Accurately mapping and aggregating SIRE groups for haplotype frequency estimation

There are multiple efforts underway to improve the quality of the estimated US population haplotype frequencies, including increasing the sample size of the frequency generating samples for some of the underrepresented populations in the NMDP registry. Only 18 of the 21 detailed SIRE frequencies are used for matching in HapLogic (Dehn et al., 2016). The excluded frequencies are AISC, SCAMB and ALANAM (Table 1) and are replaced by the corresponding broad SIRE frequency file for matching (NAM, AFA, and NAM respectively). The reason these three frequency datasets were exclude is because of the small sample size used to generate the frequencies and poor performance during matching validation. Recently generated haplotype frequencies include much larger sample sizes from NMDP registry members. Matching validation is underway to evaluate the performance of each population frequencies. Additionally, with the implementation of the new donor SIRE form in 2020, more details are being collected on Hispanic and Latino populations and other areas of the world which helps more accurate inclusion of samples for population frequency estimation and better representation of the HLA diversity in the underlying populations.

**FIGURE 1:**
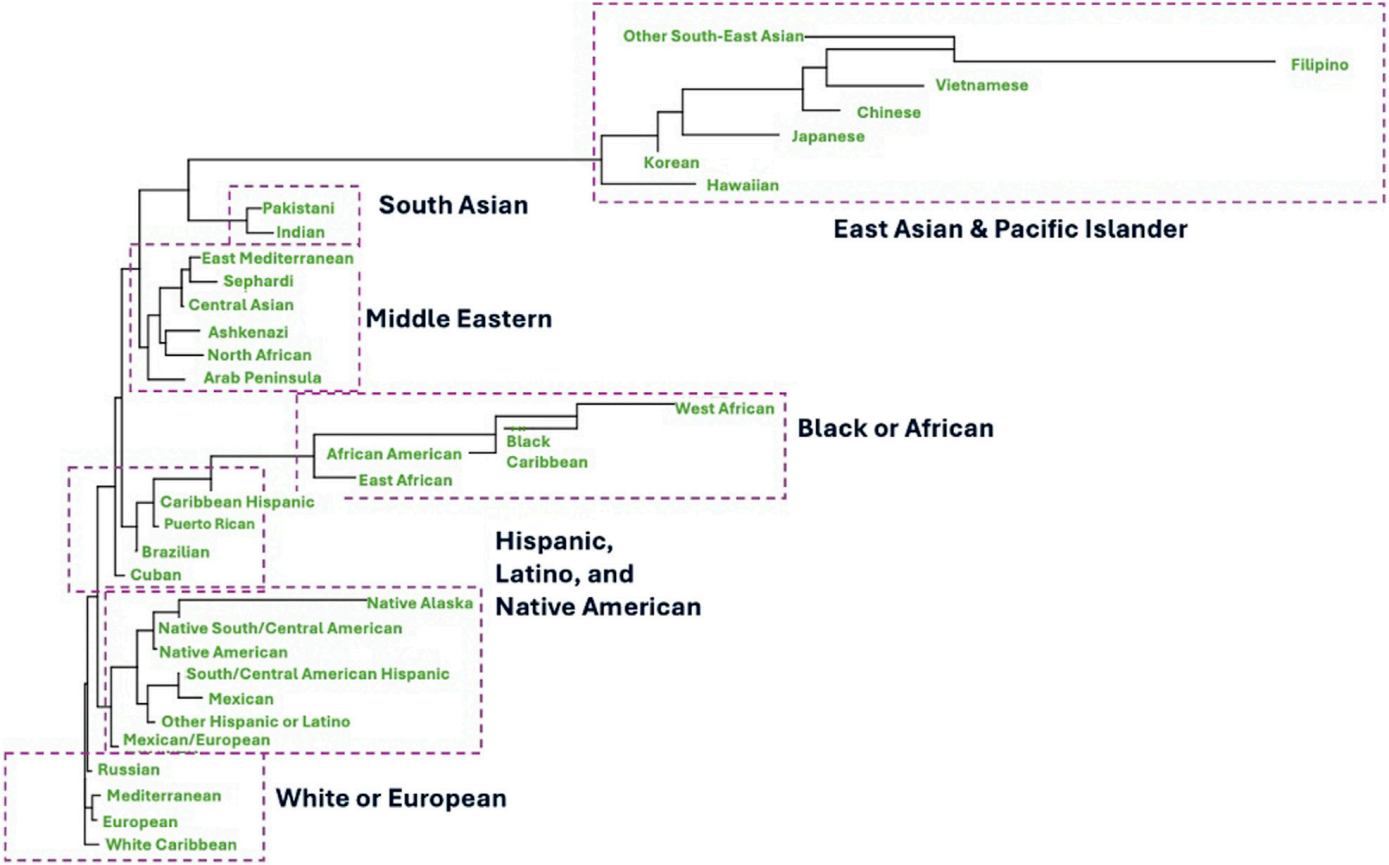
Experimental phylogenetic tree that depicts clustering of NMDP registry members based on new ancestral categories collected at recruitment using the new form implemented in 2020. Classification and tree are based on Nei’s genetic distance calculated from experimental haplotype frequencies estimated from the new experimental population groups after internal processing and adjusting for donor sample size.

Collection of more detailed donor SIRE data and the growth in the number of donors from multiple diverse populations on the NMDP registry has informed a more recent evaluation of the current operational broad and detailed SIRE categories at the NMDP (table 1). Numerous analyses are underway to evaluate the benefit of estimating dedicated population haplotype frequencies for some of the new population groups added to the new SIRE form, including Jewish, East, and West African and multiple Latino and Middle Eastern populations. This has the potential of addressing some of the existing sources of error in the frequencies, including splitting multiple combined diverse population and extracting embedded population substructure which could lead to overall improvements in the matching process (Dehn et al., 2016). Figure 1 shows some experimental results investigating the genetic distance between new member population categories based on experimental HLA haplotype frequencies estimated from these new groups. The phylogenetic tree was built using Nei’s genetic distance (Hattemer, 1982). These preliminary results demonstrate clear distinction of some populations previously unidentified on the registry or combined with other groups such as East and West African and multiple Jewish populations. Additionally, the preliminary clustering results imply that not all Hispanic and Latino populations are similar with clear genetic separation of Cuban, Brazilian, Puerto-Rican and Caribbean Hispanic groups from the Mexican population. Lastly, we observed that registry members of multiple Jewish ethnicities have more genetic proximity to Middle Eastern populations rather than European (Figure 1). These are early experimental results conducted on a limited set of NMDP registry members. However, further validation is warranted to finalize new population groups, with the goal of better distinction of member populations, improving matching and registry operations.

#### SIRE prediction from HLA data and handling mixed-ancestry

One limitation of all the methods described above used for matching, imputation and other registry operations is the use of haplotype frequencies based on single populations. None of these methods can handle data on individuals of recently admixed ancestral origin (e.g., parents belong to different continental populations). As the number of individuals of mixed ancestry grow worldwide, challenges arise for HLA matching and genotype imputation for mixed ancestry individuals. Additionally, gaps and inaccuracies in collected SIRE data form source of error for imputation and matching algorithms as well as other registry operations. We have recently implemented and extensively validated a new imputation algorithm (Israeli et al., 2023) that can impute both: HLA genotypes as well as assign a SIRE category for the input genotype based on reference data from population haplotype frequencies. This method can assign a different SIRE value for each imputed haplotype and therefore predict the single or mixed ancestry of the input HLA genotype.

## Conclusion

Self-identified race, ancestry and ethnic information is crucial for multiple healthcare applications and clinical research. This information is particularly important for the operation of volunteer stem-cell donor registries and multiple methods that benefit solid organ transplantation. SIRE information for registry members is used for multiple applications, including the estimation of haplotype frequency datasets that serve as reference data for HLA imputation and matching of donors with transplant patients. These frequencies are also utilized for modeling and projection of match rates and data-driven registry strategy planning and recruitment. Patient SIRE data is crucial for patient-driven donor recruitment, HLA imputation to resolve data gaps or ambiguities as well as identifying social determinants of health.

While recent advancements in HLA genotyping enabled wide access to affordable high-resolution HLA data, it is important to note that some ambiguities (for example, allele codes) are still reported in high-resolution data and substantial gaps still exist, particularly for newly typed loci like DPB1, DPA1 and DQA1. The majority of stem-cell registry members’ HLA data still have substantial ambiguities and data gaps and will require imputation to address these issues and enable future matching of donors and recipients beyond the 5-locus level. Current haplotype frequencies, imputation and matching methods are designed to process HLA data at the Antigen Recognition Domain (ARD) (exons 2 and 3 for class I and exon 2 for class II HLA) to conform with current matching guidelines for HCT and donor selection (Dehn et al., 2019). However, recent clinical research has demonstrated potentially favorable transplant outcomes with matching beyond the ARD (Vazirabad et al., 2019; Mayor et al., 2021). Substantial updates will need to be implemented to haplotype frequency estimation methods, imputation and matching to process HLA data at the three and four field resolution or upgrade the resolution of existing legacy data. These methods will be crucial to handle HLA data for decades to come. Lastly, current deceased donor typing for solid organ transplants are still primarily performed at the low-resolution level. If higher resolution HLA matching or multiple mismatch analyses are needed, and if high-resolution genotyping is not feasible or accessible, the application of imputation methods will still be necessary.

Numerous research initiatives have been and are still being conducted as we unravel areas of improvement in collecting and mapping this data and continue to improve matching operations for searching patients. The accuracy of SIRE data for both donors and transplant recipients can impact many aspects of the transplantation experience. While multiple challenges still exist in the collection, processing, and application of SIRE data and while other alternatives have been proposed like the use of genetic markers, this data will continue to be crucial for clinical research and multiple applications.

## Data Availability

Publicly available datasets were analyzed in this study. This data can be found here: frequency.nmdp.org.
