# Supplementary material for: Race, ethnicity, ancestry, and aspects that impact HLA data and matching for transplant
Source: Front Genet. 2024 Mar 15;15:1375352. doi: 10.3389/fgene.2024.1375352 (PMC10978785; doi:10.3389/fgene.2024.1375352)
Supplement: Supplementary file 1 [file DataSheet1.docx]

Case: HLA ambiguous genotype input to Haplostats for imputation

SIRE information: African American (AFA)

HLA genotype:


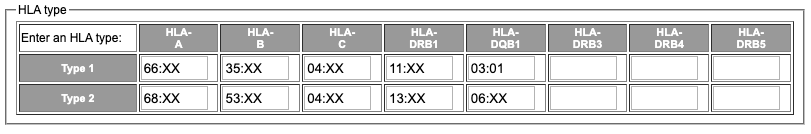


Table S1. Example input HLA genotype

Haplostats© (haplostats.org) is used as an interface for imputation to resolve ambiguities in the input HLA genotype.

Scenario 1: SIRE information is not provided and instead all populations are selected.

Result: Haplostats produces the following result:


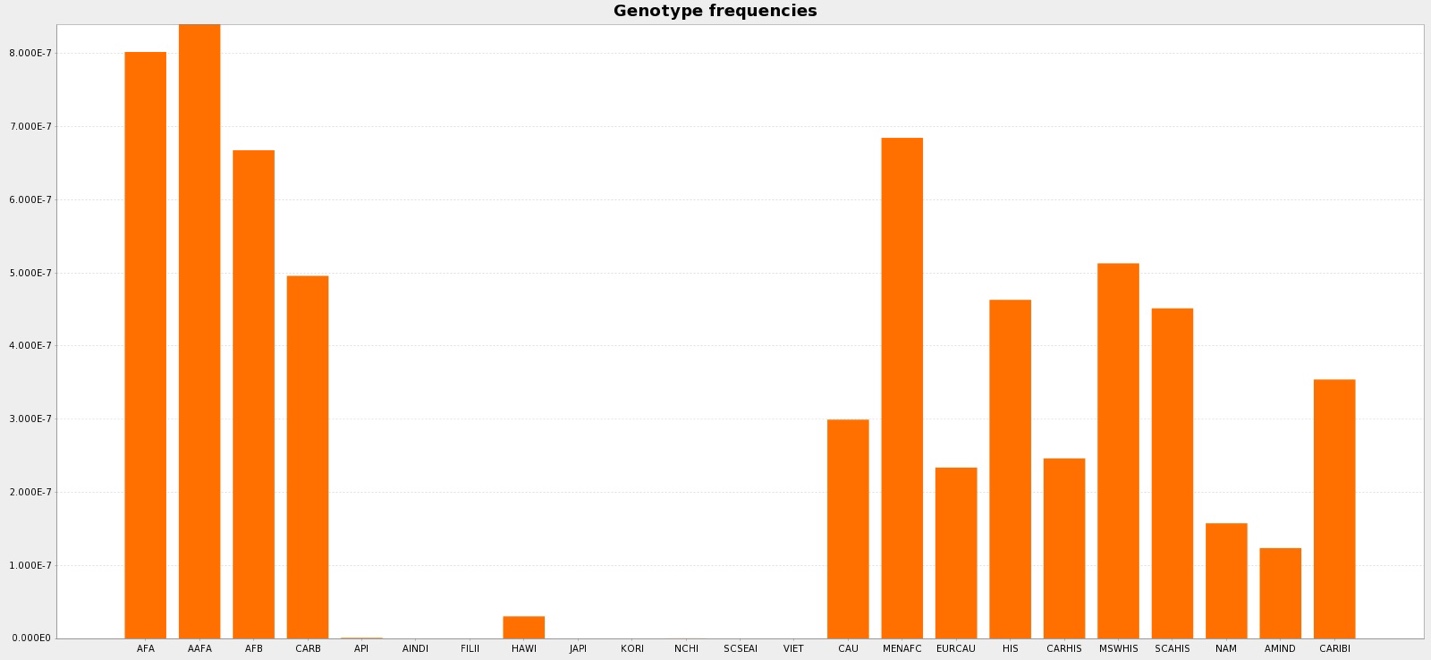


Figure S1. Haplostats bar chart showing the estimated genotype frequency of the imputed genotypes in multiple populations. Please refer to table 1 for SIRE abbreviations.


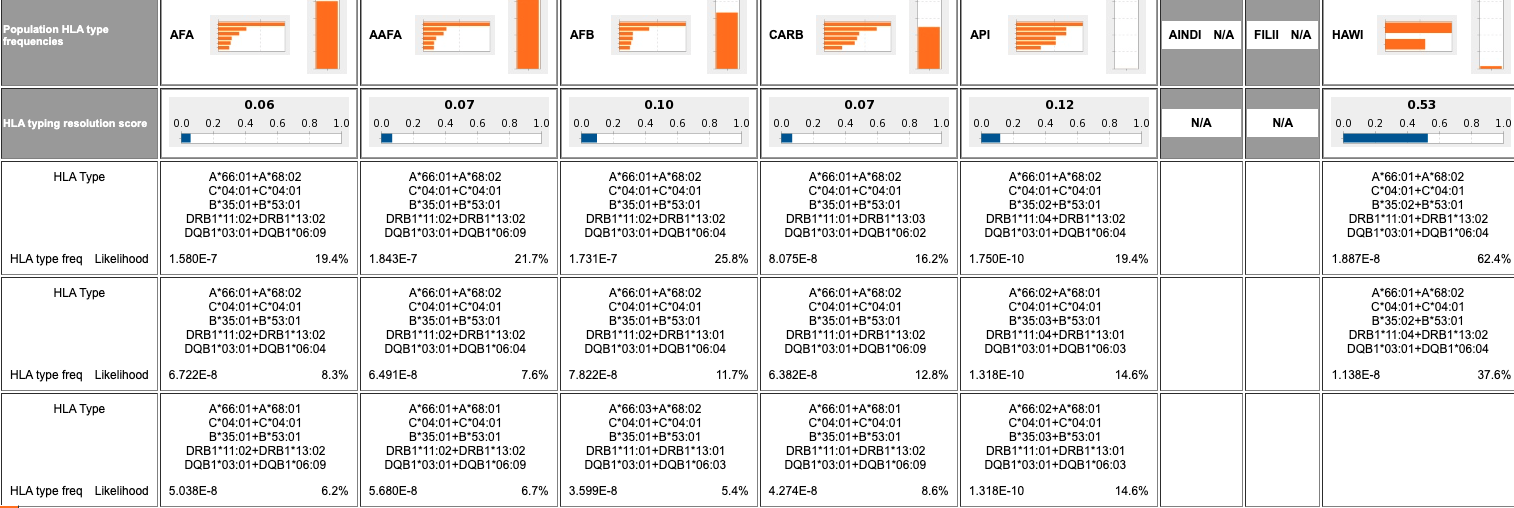


Figure S2. Genotype predictions of the input ambiguous HLA genotype in multiple populations. The SIRE information was not input to Haplostats. Haplostats allele predictions differ among populations. Please refer to table 1 for SIRE abbreviations.

Scenario 2: AFA race is input to haplostatst.org.

Result: Haplostats produces the following result:


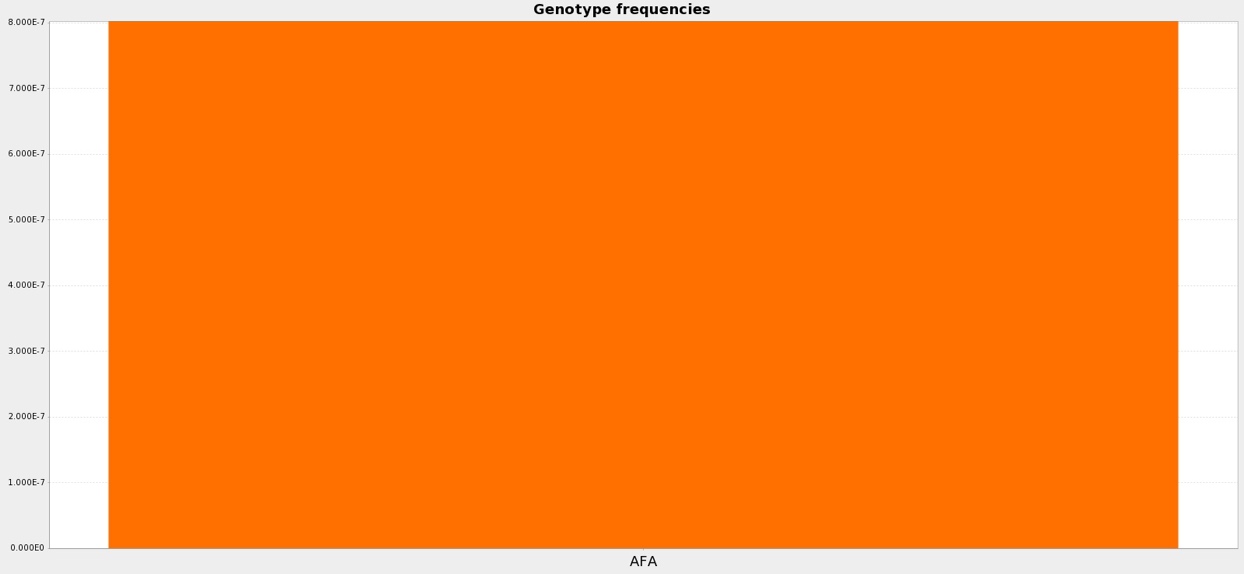


Figure S3. Haplostats bar chart showing the estimated genotype frequency of the imputed genotype in the AFA population.


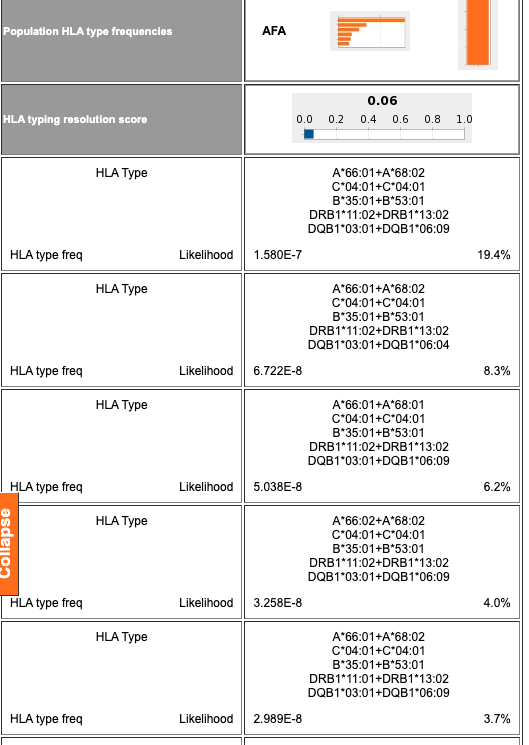


Figure S4. Genotype predictions of the input ambiguous HLA genotype in the AFA population only, based on the SIRE input to Haplostats. Haplostats allele predictions are more concordant with genotypes expected in the AFA population.
